# Development of an Effective Chain Elongation Process From Acidified Food Waste and Ethanol Into n-Caproate

**DOI:** 10.3389/fbioe.2018.00050

**Published:** 2018-04-27

**Authors:** Mark Roghair, Yuchen Liu, David P. B. T. B. Strik, Ruud A. Weusthuis, Marieke E. Bruins, Cees J. N. Buisman

**Affiliations:** ^1^Sub-department of Environmental Technology, Wageningen University and Research, Wageningen, Netherlands; ^2^Bioprocess Engineering, Wageningen University and Research, Wageningen, Netherlands; ^3^Wageningen Food and Biobased Research, Wageningen University and Research, Wageningen, Netherlands

**Keywords:** food waste, chain elongation, caproate, HRT, sludge, ethanol, sodium hydroxide

## Abstract

**Introduction:** Medium chain fatty acids (MCFAs), such as n-caproate, are potential valuable platform chemicals. MCFAs can be produced from low-grade organic residues by anaerobic reactor microbiomes through two subsequent biological processes: hydrolysis combined with acidogenesis and chain elongation. Continuous chain elongation with organic residues becomes effective when the targeted MCFA(s) are produced at high concentrations and rates, while excessive ethanol oxidation and base consumption are limited. The objective of this study was to develop an effective continuous chain elongation process with hydrolyzed and acidified food waste and additional ethanol.

**Results:** We fed acidified food waste (AFW) and ethanol to an anaerobic reactor while operating the reactor at long (4 d) and at short (1 d) hydraulic retention time (HRT). At long HRT, n-caproate was continuously produced (5.5 g/L/d) at an average concentration of 23.4 g/L. The highest n-caproate concentration was 25.7 g/L which is the highest reported n-caproate concentration in a chain elongation process to date. Compared to short HRT (7.1 g/L n-caproate at 5.6 g/L/d), long HRT resulted in 6.2 times less excessive ethanol oxidation. This led to a two times lower ethanol consumption and a two times lower base consumption per produced MCFA at long HRT compared to short HRT.

**Conclusions:** Chain elongation from AFW and ethanol is more effective at long HRT than at short HRT not only because it results in a higher concentration of MCFAs but also because it leads to a more efficient use of ethanol and base. The HRT did not influence the n-caproate production rate. The obtained n-caproate concentration is more than twice as high as the maximum solubility of n-caproic acid in water which is beneficial for its separation from the fermentation broth. This study does not only set the record on the highest n-caproate concentration observed in a chain elongation process to date, it notably demonstrates that such high concentrations can be obtained from AFW under practical circumstances in a continuous process.

## Introduction

Organic residual streams, like food waste, have great potential as alternative resource for production of fuels and chemicals because they are renewable and because they do not compete with the human food chain (Coma et al., 2017). The challenge is to convert these mixed residues into the desired products and purify them in an energy-efficient and economically viable process. An emerging technology that facilitates conversion of (derivatives of) organic residues into (precursors of) fuels and chemicals is chain elongation. Chain elongation is an anaerobic open-culture biotechnological process that converts volatile fatty acids (VFAs) and an electron donor into more valuable medium chain fatty acids (MCFAs) (Angenent et al., 2016). The conversion of VFAs into MCFAs with ethanol as electron donor is done by chain elongating micro-organisms (e.g., *Clostridium kluyveri*) that use the reverse β-oxidation pathway. In this pathway, 1 additional mole of ethanol is oxidized into acetate for every 5 chain elongation reactions (Equation 1) (Seedorf et al., 2008).

(1) $$\begin{array}{rcl} \text{Reverse }\beta & - & \text{oxidation pathway }{5C}_{x}H_{2x-1}{O_{2}}^{-}+{6C}_{2}H_{6}O\\ \to & {5C}_{x+2}H_{2x+3}{O_{2}}^{-}\;+\;C_{2}H_{3}{O_{2}}^{-}+4H_{2}O+\;H^{+}+2H_{2} \end{array}$$

VFAs can be obtained through hydrolysis and acidogenesis of organic residues. Electron donors that are suitable for chain elongation processes, such as ethanol (Steinbusch et al., 2011), hydrogen (Steinbusch et al., 2011), methanol (Chen et al., 2016b), and lactic acid (Zhu et al., 2015), can also be produced from organic residues (e.g., lignocellulosic bioethanol). Particularly, MCFAs can be used for production of aviation fuels (Harvey and Meylemans, 2014; Khor et al., 2017) and for other end-products such as solvents, lubricants, feed additives for poultry (Evans et al., 2017) and piglets (Hanczakowska et al., 2013), plastics and dyes (Angenent et al., 2016). The main advantage of chain elongation is that it is catalyzed by an anaerobic open-culture reactor microbiome (i.e., sludge). Open-culture microbiomes can tolerate mixtures of residual streams while they convert the residues under mild and non-sterile conditions. Chain elongation, therefore, does not need a chemical catalyst and proceeds under mild and non-sterile conditions. Although inhibition of competing processes is important, it is not necessary to do this by adding bioactive chemicals such as antibiotics or methanogenic inhibitors such as 2-bromoethanesulfonate (e.g., Roghair et al., 2018). As such, solid residual streams from the chain elongation process itself could be used as soil fertilizer upon composting.

MCFA production from organic residues through biomass hydrolysis, acidogenesis and chain elongation can be executed in a single-stage system (Agler et al., 2012) as well as in a two-stage system (Grootscholten et al., 2014). In a two-stage system, hydrolysis and acidogenesis are done in one stage and chain elongation in a subsequent stage. The advantage of a two-stage system over a single-stage system is that both stages can be optimized independently. Grootscholten et al. (2014) concluded that MCFA production from the organic fraction of municipal solid waste and additional ethanol in a two-stage system resulted in higher MCFA production rates and concentrations compared to a single-stage system. Another advantage of a two-stage system is that it allows easier control of the hydrogen partial pressure (*p*H_2_) in the chain elongation stage by e.g., manipulating CO_2_ loading rate (Grootscholten et al., 2014; Roghair et al., 2018). The *p*H_2_ is important because it can thermodynamically inhibit competing processes such as anaerobic oxidation of MCFAs and anaerobic oxidation of ethanol, also known as excessive ethanol oxidation (EEO; Equation 2).

(2) $$\begin{array}{rcl} \text{Excessive ethanol oxidation }\left(\text{EEO}\right)\;C_{2}H_{6}O & + & H_{2}O\to C_{2}H_{3}{O_{2}}^{-}\\ + & H^{+}+2H_{2} \end{array}$$

(3) $$\begin{array}{rcl} \text{Hydrogenotrophic methanogenesis }2H_{2} & + & 0.5CO_{2}\to 0.5CH_{4}\\ + & \;H_{2}O \end{array}$$

(4) $$\begin{array}{rcl} \text{Syntrophic ethanol oxidation }C_{2}H_{6}O\; & + & 0.5\;CO_{2}\to C_{2}H_{3}{O_{2}}^{-}\\ + & H^{+}+\;0.5\;CH_{4} \end{array}$$

Suppression of EEO is essential to make efficient use of ethanol because ethanol is a major cost factor. EEO is considered to be performed by ethanol-oxidizing microorganisms which do not perform chain elongation (Roghair et al., 2018). Earlier work demonstrated that ethanol is oxidized due to hydrogenotrophic methanogenesis (Equation 3) (Agler et al., 2014) and that the overall reaction can be referred to as syntrophic ethanol oxidation (Roghair et al., 2018) (Equation 4). By limiting the CO_2_ loading rate to a chain elongation process, EEO was reduced from 28.8 to 15.9% of total ethanol consumption (Roghair et al., 2018). No CO_2_ addition resulted in low and decreasing MCFA production rates. When working with organic residues, however, CO_2_ loading rate may be more difficult to control because CO_2_ is also a product of acidogenesis. Even though acidogenesis and chain elongation can be separated, dissolved CO_2_ could still complicate the control over the actual CO_2_ supply to the chain elongation process. In such a case, alternative strategies than CO_2_ loading rate to suppress EEO are needed. Although EEO can be beneficial for ethanol upgrading processes to n-caproate (*in situ* ethanol oxidation into acetate and subsequent chain elongation into even-numbered fatty acids), one can consider ethanol upgrading as an inefficient use of ethanol per produced MCFA (Roghair et al., 2018). Furthermore, EEO acidifies the fermentation broth and this requires extra base addition for pH correction. Because the use of both ethanol and base cause major environmental impact over the life cycle of chain elongation processes (Chen et al., 2017), their consumption should be reduced in the development of this technology.

A high MCFA concentration in chain elongation processes would be beneficial because this improves its separation from the fermentation broth (López-Garzón and Straathof, 2014). Grootscholten et al. (2014) achieved a maximum n-caproate concentration of 12.6 g/L in a two-stage MCFA production system from organic waste. Recently, considerably higher concentrations of n-caproate (>20 g/L) have been reported from chain elongation processes which were conducted in batch at near-neutral pH (Zhu et al., 2015; Liu et al., 2017). These studies suggest that such high concentrations can also be reached in a continuous chain elongation process as long as the hydraulic retention time (HRT) is long enough to allow product accumulation. The HRT, however, was also shown to influence the volumetric MCFA productivity as the highest reported MCFA production rate was achieved at a short HRT of 4 h (57 g/L/d) (Grootscholten et al., 2013a). A high MCFA productivity is desired to make effective use of the bioreactor; though MCFA production by chain elongation (62.4 g COD/L/d, Grootscholten et al., 2014) already exceeds the rate of methane production by anaerobic digestion (6.7–11.2 g COD/L/d^1^, Syngellakis, 2015). Chain elongation studies that operated at near neutral pH usually maintained an HRT shorter than 1 d (Grootscholten et al., 2013b,c, 2014; Roghair et al., 2016, 2018). To date, however, there are no studies that report the effect of a long HRT in combination with a near-neutral pH in a chain elongation process from acidified organic waste and ethanol.

The objective of this study was to develop an effective continuous biological chain elongation process from acidified food waste (AFW) and ethanol to produce n-caproate at a high concentration while EEO is limited. The effect of 2 HRTs (1 and 4 d) was compared and evaluated based on an extensive set of performance indicators including MCFA production rates, MCFA concentrations, MCFA production efficiency, substrate consumption efficiency, rate of EEO and base consumption. Finally, an outlook is given on the potential of the developed bioprocess.

## Materials and methods

### Preparation of food waste

Food waste was collected from Rotie, a recycling company in Lijnden, the Netherlands. The waste consisted of outdated food scraps and had a total solids (TS) content of 15.5 ± 0.2% (w/w), a volatile solids (VS) content of 13.8 ± 0.4% (w/w) and a sodium content of 2.7 ± 0.01 g/L. The waste was stored at −20°C until further use. Before use as fermentation feed, the waste was thawed at 4°C, diluted until ~4.0% VS (w/w) with tap water and the pH was adjusted to 5.5 with 5 M NaOH.

### First stage: hydrolysis and acidogenesis of food waste

Hydrolysis and acidogenesis of food waste was executed in a batch reactor with a working volume of 20 L as described by Chen et al. (2016a). After loading 20 L diluted food waste into the reactor, the reactor content was sparged with N_2_ for 10 min to remove oxygen. Thereafter, 200 mL inoculum (derived from a previous hydrolysis and acidogenesis run that used the same substrate and reactor configuration) and 5 mL Antifoam B Emulsion (Sigma-Aldrich, the Netherlands) were added. The reactor was then operated at 35°C, 1 atm, stirred at 44 rpm while the pH was maintained at 5.5 using a pH sensor (model QP-635-E275-S8, ProSense BV - QiS, Oosterhout, The Netherlands) and 5M NaOH. The slightly acidic pH was selected to inhibit methanogenesis (Agler et al., 2012; Ge et al., 2015). After 18 days of operation, reactor content (acidified food waste; AFW) was centrifuged (15,000 rpm for 15 min) and decanted to remove solids and sieved (1 mm) to remove floating particles (e.g., lipids). This was performed for in total four 20 L batches to generate sufficient AFW as feedstock for the chain elongation stage. The resulting centrifuged and sieved AFW from the four batches was pooled together and stored at 4°C until further use. The following compounds in the pooled AFW were measured (concentration in g/L): inorganic carbon (0.011), sodium (4.8), ethanol (0.1), butanol (0.2), acetate (8.1), propionate (1.7), isobutyrate (0.6), n-butyrate (9.3), isovalerate (0.4), valerate (0.4) and n-caproate (1.4). The mentioned organic compounds account for a chemical oxygen demand (COD) of 35 g_O2_/L. AFW had a soluble COD of 37.1 g_O2_/L (LCK 014 COD, Hach Lange GMBH, Germany).

Average VS consumption in the hydrolysis and acidogenesis stage was determined based on the mean VS content at the beginning of two batches (*n* = 6) and on the mean VS content at the end of these two batches (*n* = 6). Average NaOH consumption in the hydrolysis and acidogenesis stage was determined based on the difference between the mean sodium content of diluted food waste (*n* = 3) and the mean sodium content of AFW (*n* = 3).

### Seconds stage: chain elongation of acidified food waste and ethanol

Chain elongation of AFW and ethanol was performed in one single process using a continuously stirred anaerobic reactor as described by Roghair et al. (2016). In short, a continuous reactor with a working volume of 1 L was operated at 30°C, 1 atm, stirred at 100 rpm while the pH was maintained at 6.8 using a pH sensor (Applisens model Z001023551, Schiedam, The Netherlands) and 2M NaOH. Gaseous CO_2_ was supplied with a mass-flow controller (Brooks Instruments 5850S, the Netherlands) at 1 L_CO2_/L/d. The reactor was started with a synthetic medium that contained 13.8 g/L propionic acid (≥99.5%, Sigma-Aldrich) and 32.2 g/L ethanol (Absolute, VWR). These starting conditions have formerly shown to induce formation of granular sludge (Roghair et al., 2016) and can also be used to distinguish the carbon flux of ethanol upgrading from VFA upgrading (Roghair et al., 2018). The composition of other components (salts, yeast extract, vitamins and trace elements) were as previously described (Roghair et al., 2016). The reactor was inoculated in batch mode on day 1 with 50 mL chain elongation sludge from a previous run; the inoculum contained chain elongating micro-organisms, ethanol oxidizers and hydrogenotrophic methanogens (Roghair et al., 2018). On day 9, the reactor operation mode was set from batch to continuous with an HRT of 4 d. From day 19 onwards, the reactor was fed with AFW to which 32.2 g/L ethanol was added. On day 58, the HRT was set from 4 to 1 d. On day 103, the HRT was set back from 1 to 4 d.

Liquid samples were taken from the reactor content and from the influent tank 1-3 times per week. Gas samples were taken from the headspace 1-3 times per week. The reactor was assumed to be in steady state when n-caproate production rates were similar (with a maximum relative standard deviation of 16%) over a period of at least 7 HRTs. Average concentrations and rates and their corresponding standard deviations were based on at least nine measurements within a steady state. Average NaOH consumption in the chain elongation stage was determined based on the difference between average sodium concentration in the effluent (*n* = 3 different samples during a steady state) and on the average sodium concentration in the influent tank (*n* = 3 different samples during a steady state).

### Analytical procedures

Alcohols (C2-C6) and fatty acids (C2-C8) were analyzed by gas chromatography using an Agilent 7890B (USA), equipped with HP-FFAP capillary column (l = 25 m, ID = 0.32 mm, film = 0.5 μm). 1 μl from a diluted sample was injected into a liner with glass wool at 250°C. Vaporized compounds entered the column, along with helium as carrier gas, with a flow rate of 1.25 mL/min (first 3 min) and 2 mL/min (until the end of the run). The oven temperature program was as follows: 60°C for 3 min; 21°C/min up to 140°C; 8°C/min up to 150°C and constant for 1.5 min; 120°C/min up to 200°C and constant for 1.25 min; 120°C/min up to 240°C and constant for 3 min. Compounds were detected with a flame ionization detector at 240°C, fed with 30 mL/min hydrogen and 400 mL/min air.

Gaseous compounds (N_2_, H_2_, CO_2_, CH_4_, and O_2_) were analyzed by gas chromatography using an Agilent Varian CP4900 μGC (USA) equipped with a thermal conductivity detector and two parallel columns: a Mol Sieve 5A PLOT column (l = 10 m, ID = 0.32 mm, film = 0.15 μm) and a PoraPlot U column (l = 10 m). The oven temperature was 80°C for the Mol Sieve 5A PLOT column and 65°C for the PoraPLOT U column. The carrier gas was argon and had a flow rate of 1.47 mL/min.

Sodium was measured by ion chromatography using a Metrohm Compact IC Flex 930 (Schiedam, the Netherlands) equipped with a pre-column (Metrohm Metrosep RP 2 Guard/3.6), a cation column (Metrosep C 4-150/4.0) and a conductivity detector. The mobile phase was 3mM nitric acid.

TS, VS, and VSS were determined following Standard Methods (APHA, 1998). The filter for VSS measurements (Whatman GF/F 0.7 μm) was preheated at 450°C prior to filtration. Inorganic carbon was measured using a total organic carbon analyser (Shimadzu TOC-VCPH, Japan).

### Mathematical expressions

The volumetric production or consumption rate of aqueous compounds is based on the difference between effluent concentration and influent concentration divided by the HRT:

Rate [g/L/d] = (effluent concentration [g/L] – influent concentration [g/L]) / HRT [d]

Excessive ethanol oxidation is the difference between total ethanol consumption and ethanol consumption through the reverse β-oxidation pathway:

Excessive ethanol oxidation (EEO) [g/L/d] = rate total ethanol consumption [g/L/d] – rate ethanol consumption through the reverse β-oxidation pathway [g/L/d] (Roghair et al., 2018)

Ethanol consumption through the reverse β-oxidation pathway (g/L/d) = ethanol use for elongation of fatty acids through the reverse β-oxidation pathway (g/L/d) + ethanol oxidation into acetate through the reverse β-oxidation pathway (g/L/d)

Ethanol use for elongation of fatty acids through the reverse β-oxidation pathway (g/L/d) = (rate n-butyrate [mmol/L/d] + rate valerate [mmol/L/d] + 2· rate n-caproate [mmol/L/d] + 2· rate n-heptanoate [mmol/L/d] + 3 · rate n-caprylate [mmol/L/d]) · 46.05 / 1000

Ethanol oxidation into acetate through the reverse β-oxidation pathway (g/L/d) = Ethanol use for elongation of fatty acids through the reverse β-oxidation pathway (g/L/d) · 0.2

Selectivity is defined as product produced relative to substrates consumed on an electron basis (Grootscholten et al., 2014):

Selectivity [mol e %] = product formation rate [mol e/L/d] / total substrate consumption rate (mol e /L/d) · 100

Selectivity values that are based on a carbon basis are reported in the supplementary material but are not presented and discussed in the results and discussion section.

Substrate consumption efficiency is defined as substrate consumed relative to the organic loading rate on an electron basis:

Substrate consumption efficiency [mol e %] = (|substrate consumption rate [mol e/L/d]| / organic loading rate [mol e/L/d] · 100

Product production efficiency is defined as product produced relative to the organic loading rate on an electron basis

Product production efficiency [mol e %] = product formation rate [mol e/L/d] / organic loading rate [mol e/L/d] · 100

## Results

### Higher MCFA concentrations and selectivities at long HRT than at short HRT

**Figure 1:**
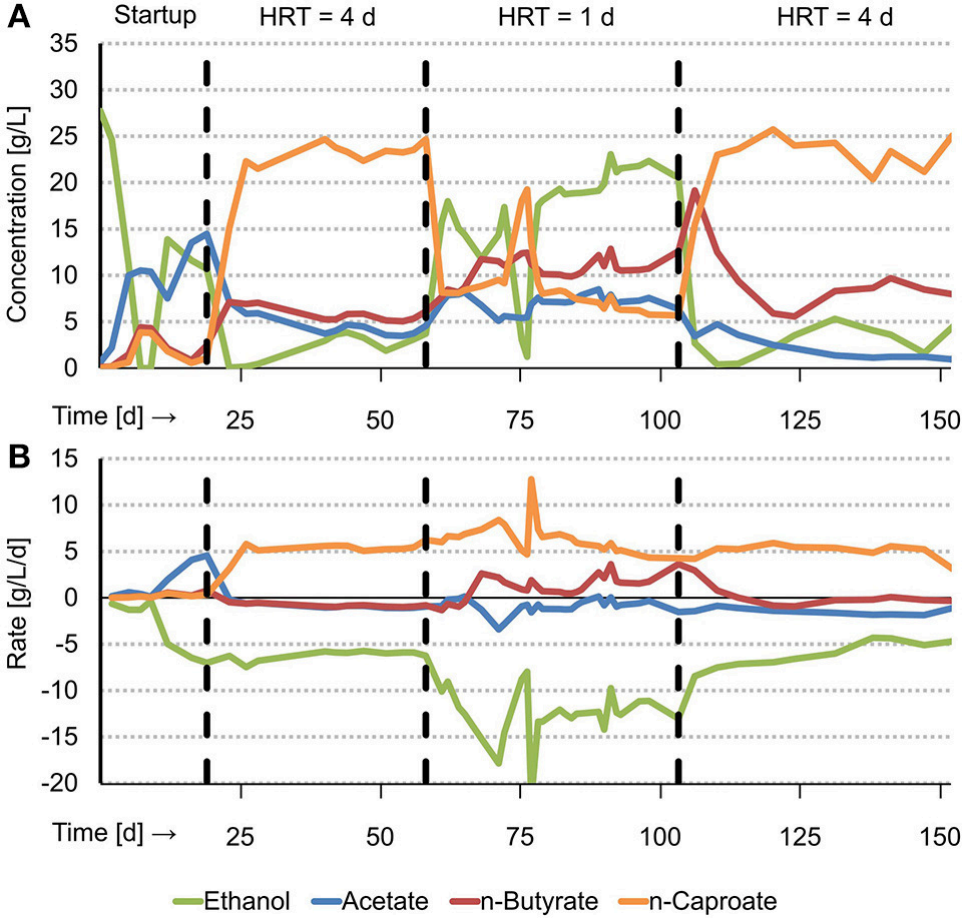
Graphical summary of experimental results with concentrations over time **(A)** and net production and consumption rates over time **(B)**. T = 30°C, pH = 6.8, V = 1 L, CO_2_ loading rate = 1.0 L_CO2_/L/d.

Acidified food waste (AFW) and ethanol were fed to a continuous biological chain elongation process, resulting in production of MCFAs (n-caproate, isocaproate, n-heptanoate and n-caprylate). n-Caproate, the dominant MCFA, was produced (5.5 ± 0.4 g/L/d) at a high steady state concentration of 23.4 ± 1.0 g/L. This was observed at long HRT (4 d) from day 28 through day 58 (Figure 1). After the HRT was decreased from 4 to 1 d (short HRT), another steady state was observed from day 79 through day 103. Here, n-caproate was produced at a similar rate (5.6 ± 0.9 g/L/d) but at a lower concentration (7.1 ± 0.9 g/L). On day 103, the HRT was increased from 1 to 4 d. Again, n-caproate was continuously produced at a high steady state concentration (23.2 ± 1.9 g/L). This was observed from day 114 through day 147. The maximum n-caproate concentration was 25.7 g/L on day 120. Reactor performance of the first steady state at long HRT was similar as the reactor performance of the second steady state at long HRT. This shows that the effect of HRT on reactor performance is reversible. From here on, however, “long HRT” refers to the first steady state (day 28–58). Mean steady state rates, concentrations and selectivities of all identified substrates and products at long and at short HRT are reported in Tables S1 and S2 respectively. A summary of reactor performance indicators and properties of the steady states at both long and short HRT are reported in Table 1 for comparison.

**Table 1:** Performance indicators and properties of the chain elongation process at long and at short HRT.

**Performance indicator**	**Unit**	**Long HRT [4 d]**	**Short HRT [1 d]**
**STEADY STATE CHARACTERISTICS**			
Steady state interval	d	28–58	79–103
Number of HRTs	–	7.5	24.3
**PRODUCTS**			
n-Caproate concentration	g/L	23.4 ± 1.0	7.1 ± 0.9
n-Caproate rate	g/L/d	5.5 ± 0.4	5.6 ± 0.9
n-Caproate selectivity	mol e %	76.5 ± 5.0	44.6 ± 7.8
MCFA selectivity	mol e %	81.6 ± 5.0	46.3 ± 8.0
Methane rate	mmol/L/d	12.8 ± 2.6	43.8 ± 2.5
**SUBSTRATES**			
Ethanol loading rate	g/L/d	6.1 ± 0.2	30.6 ± 1.3
Ethanol concentration	g/L	2.8 ± 1.1	20.1 ± 1.6
Ethanol rate	g/L/d	−6.0 ± 0.3	−12.3 ± 1.1
EEO	g/L/d	0.9 ± 0.4	5.6 ± 1.4
EEO	% of total ethanol use	14.7 ± 5.5	45.0 ± 9.7
Acetate rate	g/L/d	−1.0 ± 0.2	−0.8 ± 0.5
Propionate rate	g/L/d	−0.3 ± 0.0	−0.7 ± 0.1
n-Butyrate rate	g/L/d	−0.9 ± 0.1	1.6 ± 1.1
**SUBSTRATE TO PRODUCT CONVERSIONS**			
Consumed VFA per produced MCFA	mol C/mol C	0.29 ± 0.04	0.20 ± 0.07
Consumed Ethanol per produced MCFA	mol C/mol C	0.87 ± 0.07	1.83 ± 0.31
**CONSUMPTION/PRODUCTION EFFICIENCY**			
Ethanol consumption efficiency	mol e %	98.6 ± 5.4	40.3 ± 3.5
VFA consumption efficiency	mol e %	45.8 ± 6.9	7.2 ± 2.3
n-Caproate production efficiency	mol e %	58.7 ± 2.9	12.8 ± 2.1
MCFA production efficiency	mol e %	62.6 ± 2.9	13.3 ± 2.2
**NaOH USE**			
Sodium concentration in influent [AFW]	g/L	4.9 ± 0.1	4.8 ± 0.1
Sodium concentration in reactor	g/L	9.1 ± 0.5	7.0 ± 0.0
Consumed NaOH per produced MCFA	mol/mol	0.92 ± 0.04	1.93 ± 0.31
**VSS**			
VSS concentration	g/L	0.34 ± 0.23	0.33 ± 0.03
VSS rate	g/L/d	0.02 ± 0.05	0.18 ± 0.33
Growth rate	g/g/d	0.07 ± 0.16	0.54 ± 1.00

At long HRT, n-heptanoate and n-caprylate were both produced at a low rate (~0.15 g/L/d) and concentration (~0.6 g/L) compared to n-caproate. At short HRT, however, these MCFAs were produced at insignificant amounts (<0.1 g/L). Isocaproate was produced in trace amounts at both long and short HRT. The selectivity of MCFAs was 81.6 mol e % at long HRT and 46.3 mol e % at short HRT. The remaining consumed carbon ended up as VSS (biomass), VFAs, methane, propanol, and unidentified products as given in Tables S1 and S2. The MCFA productivity at both long and short HRT (~12.5 g COD/L/d) was somewhat higher compared to the typical methane productivity in an anaerobic digester (6.7–11.2 g COD/L/d^1^, Syngellakis, 2015).

### Excessive ethanol oxidation, methane production and NaOH consumption

Long HRT did not only result in higher MCFA concentrations and selectivities compared to short HRT, it also resulted in less EEO and in less sodium hydroxide (NaOH) consumption per MCFA produced. EEO occurred at a 6.3 times lower rate at long HRT (0.9 ± 0.4 g/L/d) than at short HRT (5.6 ± 1.4 g/L/d). This process was also relative to the total ethanol consumption rate lower at long HRT (14.7 ± 5.5%) than at short HRT (45.0 ± 9.7%). Methane production was 3.4 times lower at long HRT (12.8 ± 2.6 mmol/L/d) than at short HRT (43.8 ± 2.5 mmol/L/d). A previous study showed that hydrogenotrophic methanogenesis and EEO are coupled and that the overall reaction can be referred to as syntrophic ethanol oxidation (Roghair et al., 2018). In syntrophic ethanol oxidation, the stoichiometric ratio between methane production and ethanol oxidation is 0.5 mol/mol (Equation 4). The present study shows a similar ratio at long HRT (0.7 ± 0.3) and at short HRT (0.4 ± 0.1) which implies that EEO, like in the previous study, was a result of syntrophic ethanol oxidation. Less EEO also led to fewer NaOH consumption for pH correction because EEO is an acidifying process that releases a proton (Equation 2). NaOH consumption per produced MCFA was two times lower at long HRT (0.92 ± 0.04 mol_NaOH_/mol_MCFA_) than at short HRT (1.93 ± 0.31 mol_NaOH_/mol_MCFA_). These results not only show that EEO can be limited at long HRT but also that consumption of NaOH is hereby reduced.

### Consumption of VFAs and ethanol

Acetate, propionate and ethanol were consumed at both long and short HRT. However, whereas butyrate was consumed at long HRT (−0.9 ± 0.1 g/L/d), it was produced at short HRT (1.6 ± 1.1 g/L/d). This resulted in a lower n-butyrate concentration in the reactor at long HRT (5.6 ± 0.6 g/L) than at short HRT (10.8 ± 1.0 g/L). A net consumption of n-butyrate instead of production indicates that caproate production is more efficient in ethanol-use. This is because n-butyrate consumption (i.e., elongation) requires less ethanol than acetate elongation to caproate or ethanol upgrading. Whereas ethanol upgrading requires 3 moles of ethanol to produce 1 mole of n-caproate, VFA upgrading requires only 1.2 moles of ethanol from n-butyrate or 2.4 moles of ethanol from acetate (Roghair et al., 2018). Indeed, the chain elongation process at long HRT was more efficient in ethanol-use than at short HRT. Approximately two times less ethanol was consumed per produced MCFA at long HRT (0.87 ± 0.07 mol C/mol C) than at short HRT (1.83 ± 0.31 mol C/mol C). The VFA consumption per produced MCFA at both long (0.29 ± 0.04 mol C/mol C) and short HRT (0.20 ± 0.07 mol C/mol C) were found to be similar.

The concentration of ethanol in the reactor (and thus also in the effluent) was much lower at long HRT (2.8 ± 1.1 g/L) than at short HRT (20.1 ± 1.6 g/L). The ethanol consumption efficiency, therefore, defined as consumed ethanol relative to supplied ethanol, was higher at long HRT (98.6 ± 5.4 mol e %) than at short HRT (40.3 ± 3.5 mol e %). A high ethanol consumption efficiency (or a low ethanol concentration in the effluent) is desired because any unconsumed ethanol requires an additional recovery or treatment step after the chain elongation stage which makes the overall process more expensive. The VFA consumption efficiency was also higher at long HRT (45.8 ± 6.9 mol e %) than at short HRT (7.2 ± 2.3 mol e %). Although VFAs are not as costly as ethanol, it is evident that a higher VFA consumption efficiency is preferred because the remaining VFAs (e.g., after selective extraction of the MCFAs) also have to be recovered or treated with a waste water treatment system.

### VSS

The mean VSS concentration in the reactor at long HRT (0.34 ± 0.23 g/L) was similar compared to the mean VSS concentration in the reactor at short HRT (0.33 ± 0.03 g/L). These reactor concentrations were in the same order of magnitude as the VSS concentrations in the effluent (0.43 ± 0.32 g/L at long HRT and 0.35 ± 0.20 g/L at short HRT), implying that the reactor was ideally stirred with no biomass retention (i.e., CSTR). Formation of granular sludge, however, was observed like in the earlier experiment with the same set-up while using a synthetic medium (Roghair et al., 2016). The earliest observation of granules (by eye visible) was on day 82, at short HRT. Granules disappeared within a few days after the HRT was increased on day 103. Because the formation of granular sludge coincided with high-rate syntrophic ethanol oxidation (at short HRT) it is likely that this syntrophic process attributed to sludge granulation. Syntrophic processes may benefit from granulation because granules could facilitate a more efficient interspecies hydrogen transfer due to the decreased intermicrobial distances (Kouzuma et al., 2015).

The mean VSS concentration in the influent was 0.25 ± 0.06 g/L. From the mentioned values the VSS production rate and VSS specific growth rate were calculated (Table 1). Although one could expect a four times lower growth rate at long HRT (0.07 ± 0.16 g/g/d) than at short HRT (0.54 ± 1.0 g/g/d), there was no significant difference due to the large standard deviations.

## Discussion

### Continuous n-caproate production at a high concentration from acidified food waste

In this study, an effective two-stage MCFA production process from food waste and ethanol was developed. The microbiome in the second stage (chain elongation) was able to continuously produce n-caproate at 23.4 ± 1.0 g/L while EEO was limited to 14.7 ± 5.5% of total ethanol consumption. This was achieved at long HRT (4 d) and at near-neutral pH (6.8) but without in-line product extraction. Thus, a long HRT was shown to be effective for this specific waste stream. The n-caproate production rate was similar for both long and short HRT (~5.5 g/L/d) and was much lower compared to the highest reported n-caproate production rate to date (55.8 g/L/d) (Grootscholten et al., 2013a). This high production rate, however, occurred at a substantially lower n-caproate concentration (9.3 g/L) than obtained in the present study. An overview of comparable studies that reported high n-caproate concentrations and/or rates using open cultures is shown in Table 2.

**Table 2:** Overview of comparable studies that report high n-caproate concentrations and/or rates using open cultures.

**Reactor type**	**Substrate(s)**	**pH**	**HRT**	**n-Caproate**			**References**
				**Concentration [g/L]**	**Rate [g/L/d]**	**Selectivity [mol e%]**	
Continuously stirred anaerobic reactor	AFW and ethanol	6.8	4 d	23.4	5.5	76.5	This study
Continuously stirred anaerobic reactor	AFW and ethanol	6.8	1 d	7.1	5.6	44.6	This study
Batch reactor	Lactate	~6.5	N.A.	23.4	1.1	81.4	Zhu et al., 2015
Batch reactor	Acetate and ethanol	~6.5	N.A.	21.1	N.D.	65.0	Liu et al., 2017
Continuous upflow anaerobic filter	Acetate and ethanol	6.5–7.2	4 h	9.3	55.8	~78.0	Grootscholten et al., 2013a
Continuous upflow anaerobic filter	Acidified food/garden waste and ethanol	6.5–7.0	11 h	12.6	26.0	72.0	Grootscholten et al., 2014
Continuous upflow anaerobic filter	Acetate and ethanol	6.5–7.0	17 h	11.1	15.7	85.0	Grootscholten et al., 2013c

*N.A., Not applicable*.

*N.D., Not determined*.

A high n-caproate concentration is beneficial for its separation from the fermentation broth. The obtained n-caproate concentration in the present study is more than twice as high as the maximum solubility of the undissociated form of n-caproate, n-caproic acid, in water (~10.8 g/L, Yalkowsky et al., 2016). Thus, in theory, the high n-caproate concentration allows it to be separated from the effluent by phase separation after lowering the pH to 4.9 or lower. A recent study, executed by Zhu et al. (2015), also reported a high n-caproate concentration (23.4 g/L) using an anaerobic reactor microbiome. The n-caproate was produced from lactate as the sole carbon source in a batch process using an inoculum that was derived from mature pit mud, a microbiome used for the production of Chinese strong-flavored liquor. Their process reached a higher n-caproate selectivity (81.4 mol e %) than the process in the present study at long HRT (76.5 mol e %). However, the process in the present study achieved a slightly higher MCFA selectivity (81.6 mol e %) because it also produced other MCFAs (isocaproate, n-heptanoate and caprylate).

Liu et al. (2017) also reported a high n-caproate concentration (21.2 g/L) from ethanol and acetate using an anaerobic reactor microbiome. They were able to produce this in a batch process upon addition of biochar and 2-bromoethanesulfonate (Liu et al., 2017). The maximum n-caproate selectivity was lower (65.0 mol e %) than the maximum n-caproate selectivity in the present study (76.5 mol e %).

The studies by Zhu et al. (2015), and by Liu et al. (2017) already showed that high n-caproate concentrations (>20 g/L) can be reached using anaerobic reactor microbiomes. However, they were using synthetic media and batch systems. As such, the present study does not only show that such high concentrations can be obtained from organic residues, it also shows that this can be obtained in a continuous process and without the use of bioactive compounds such as 2-bromoethanesulfonate. The organic residue that was used, food waste, is a suitable substrate for MCFA production not only because such conversion was recently subjected to a life cycle assessment (Chen et al., 2017) but also because it is currently being developed to a demonstration factory, processing ~40 ton/day, by ChainCraft in Amsterdam, the Netherlands.

### Why was reactor performance so much better at long HRT than at short HRT?

The performance of the chain elongation process was far better at long HRT than at short HRT. A long HRT did not only result in a higher concentration of MCFAs, it also led to a lower rate of syntrophic ethanol oxidation, a net n-butyrate consumption instead of production, and to less NaOH consumption for pH correction. Why was reactor performance so much better at long HRT?

The difference in MCFA concentration can be explained as follows: at long HRT, the microbiome had sufficient time to accumulate MCFAs while these products were washed out (as effluent) at a relatively low rate. This resulted in a high caproate concentration (23.4 ± 1.1 g/L). Vice versa, at short HRT, the microbiome had little time to accumulate MCFAs while these products were washed out at a relatively high rate. This resulted in a considerably lower caproate concentration (7.1 ± 0.9 g/L). Because production of n-caproate occurred at the same volumetric production rate at both long and short HRT (~5.5 g/L/d), it is a logical consequence that n-caproate reached a higher concentration at long HRT than at short HRT. The high n-caproate concentration could also be achieved because the process was not limited by availability of substrates as sufficient ethanol (2.8 ± 1.1 g/L), acetate (4.2 ± 0.8 g/L) and n-butyrate (5.6 ± 0.6 g/L) was observed in the reactor. At short HRT, however, substantially more ethanol (20.1 ± 1.6 g/L), acetate (7.3 ± 0.5 g/L) and n-butyrate (10.8 ± 1.0 g/L) was observed but no higher MCFA production rates. This seems to be limited by the biomass concentration or by the high (i.e., inhibitory) ethanol concentration (Lonkar et al., 2016).

Syntrophic ethanol oxidation was more limited at long HRT than at short HRT. Two explanations can be given: firstly, the low ethanol concentration at long HRT may have resulted in a low rate of EEO. Vice versa, the high ethanol concentration at short HRT may have resulted in a high rate of EEO. However, a previous chain elongation study showed that ethanol loading rate and ethanol concentration does not substantially influence reactor performance as long as ethanol is not depleted (Roghair et al., 2018). Secondly, the high MCFA concentration may have caused an inhibitory effect on (one of) the involved competing syntrophs (i.e., ethanol oxidizers and hydrogenotrophic methanogens). It is known that undissociated MCFAs are toxic to microorganisms because they can damage the cell membrane (Royce et al., 2013). The average undissociated MCFA concentration at long HRT (at pH 6.8) was 0.27 g/L. Ge et al. (2015) determined that chain elongation proceeds until a toxic limit of 0.87 g/L undissociated n-caproic acid is reached (Ge et al., 2015) although recent work demonstrated chain elongation activity up to 1.46 g/L undissociated n-caproic acid (Andersen et al., 2017). Because chain elongating microorganisms were producing MCFAs at ~5.8 g/L/d in the present study, evidently these organisms were not rigorously inhibited by the undissociated MCFAs. It is well possible, however, that these undissociated MCFAs (at 0.27 g/L) were selectively inhibitory to either ethanol oxidizers or hydrogenotrophic methanogens. This would explain the limited rate of syntrophic ethanol oxidation at long HRT compared to a short HRT while chain elongation could proceed at a similar rate at both HRTs. It is also possible that the dissociated form of n-caproate was selectively inhibitory to one of the syntrophs. This means that dissociated n-caproate (i.e., the conjugate base) becomes toxic to ethanol oxidizers or hydrogenotrophic methanogens at a concentration around 20 g/L.

The data is not consistent to point out whether hydrogenotrophic methanogens or ethanol oxidizers were more inhibited at long HRT: whereas the first steady state at long HRT (day 28 to day 58) had a *p*H_2_ below the detection limit of the gas chromatograph (<0.1%), the second steady state at long HRT (day 138–147) had a *p*H_2_ of up to 30%. This means that the data from the first steady state suggests that ethanol oxidizers were more inhibited whereas data from the second steady state suggests that hydrogenotrophic methanogens were more inhibited. To what extent n-caproic acid and n-caproate is toxic to hydrogenotrophic methanogens and ethanol oxidizers could be elucidated in further studies. In any way, irrespective how syntrophic ethanol oxidation was limited, it is clear from the results that longer HRTs should be applied in chain elongation processes at near-neutral pH to allow n-caproate accumulation and to limit the rate of syntrophic ethanol oxidation. This limited rate of syntrophic ethanol oxidation results in a more efficient use of ethanol, more VFA consumption and in less base addition for pH control and as such, in a more effective chain elongation process.

### Methanogenic UASB sludge can acclimate into a reactor microbiome that is able to produce n-caproate at high concentrations

This study can be compared with a previous study (Grootscholten et al., 2014). Both studies focused on two-stage MCFA production from organic residues and ethanol using anaerobic reactor microbiomes. In the previous study, a continuous chain elongation process was operated at similar pH (6.5–7.0) but at shorter HRT than in the present study (11 h instead of 1 and 4 d). This resulted in a lower maximum n-caproate concentration (12.6 g/L) and selectivity (72.0 mol e %). Besides, n-butyrate was produced instead of consumed, indicating that the process was not efficient in ethanol use. A hypothesis is that the process in the previous study could have performed better, including a high n-caproate concentration, if both the HRT and the ethanol concentration in the influent were increased. Of course, the origin of the inoculum may also have an effect on reactor performance because this determines which pathways or microorganisms are introduced and to what extent the initial microbiome is acclimated. The inoculum used by Grootscholten et al. (2014) and also the inoculum used in the present study were both eventually derived from a study executed by Steinbusch et al. (2011), who used granular sludge from an upflow anaerobic sludge blanket (UASB) reactor treating brewery wastewater (Steinbusch et al., 2011). This shows that high n-caproate concentrations can be obtained using various types of inocula and not only with mature pit mud (e.g., Zhu et al., 2015) or with mesophilic sludge from an anaerobic reactor treating paper mill wastewater (e.g., Liu et al., 2017). Under the right circumstances (e.g., as described in this study), UASB sludge from a methanogenic reactor will likely acclimate within a matter of weeks to a reactor microbiome that is able to produce n-caproate at high concentrations (>20 g/L).

### Consumption of food waste, ethanol and base in the overall two-stage system

In this study, MCFAs were produced from food waste and ethanol using a two-stage system. In the hydrolysis and acidogenesis stage, part of the food waste was converted into VFAs while some MCFAs were also produced. In the chain elongation stage, a part of the VFAs from the hydrolysis and acidogenesis stage were converted with additional ethanol into MCFAs while some ethanol was also used for ethanol upgrading. Both stages required NaOH addition to keep the pH constant in the reactors. Based on the results of this study it was possible to calculate how much food waste (expressed as g VS), ethanol and NaOH would be consumed to yield 1,000 g MCFAs. This was done for two scenarios; at long and at short HRT in the chain elongation stage. Equations are shown in Table S3. Parameters that were used as input for the equations are shown in Table S4 and were based on:

- Observed conversions and average NaOH consumption in the hydrolysis and acidogenesis stage (VFA yield on VS, MCFA yield on VS, consumed NaOH per consumed VS).
- Observed steady state conversions and NaOH consumption in the chain elongation stage (consumed VFAs per produced MCFAs, consumed ethanol per produced MCFAs, consumed NaOH per consumed ethanol).
- Average MCFA composition in the chain elongation stage (average carbon atoms per produced MCFAs, average molar weight of produced MCFAs).

**Figure 2:**
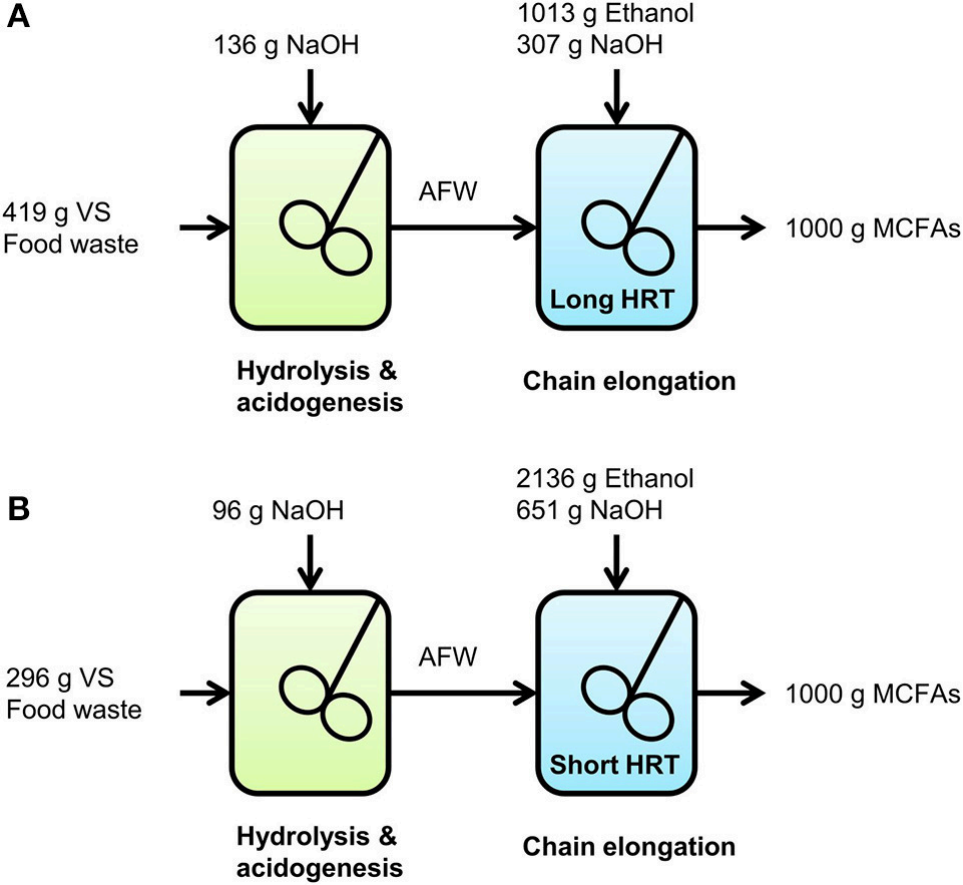
Graphical representation on how much food waste (g VS), ethanol and NaOH would be consumed to yield 1,000 g MCFAs in the two-stage system at long HRT **(A)** and at short HRT **(B)** in the chain elongation stage.

A graphical representation on how much food waste (in g VS), ethanol and NaOH would be consumed to yield 1,000 g MCFAs at long and short HRT in the chain elongation stage is shown in Figure 2.

To produce 1,000 g MCFAs, the two-stage system with a long HRT in the chain elongation stage would consume 53% less ethanol, 41% less NaOH and 42% more VS compared to a two-stage system with a short HRT in the chain elongation stage. This does not only show that a long HRT in the chain elongation stage result in a more efficient consumption of ethanol and base in the chain elongation stage itself, it also shows that base consumption is hereby reduced in the overall two-stage system. Unfortunately, such quantification on waste-use and chemical-use is not common in chain elongation studies to date.

Addition of ethanol could be minimized or even completely avoided by steering the hydrolysis and acidogenesis stage to MCFAs and/or lactate production. In this study, for example, 1.6 g/L n-caproate was produced in the hydrolysis and acidogenesis stage without addition of an external electron donor. Lim et al. (2008) also demonstrated production of n-caproate (up to 5 g/L) from food waste without an external electron donor (Lim et al., 2008). Xu et al. (2017) demonstrated conversion of acid whey waste into MCFAs via lactate (Xu et al., 2017) using a two-stage system; also without an external electron donor. This shows that the effectiveness of MCFA production processes from diverse organic waste streams can be further improved by optimizing the hydrolysis and acidogenesis stage. Studies should also report on how much base or acid or electricity was used for pH control for a better comparison in terms of effectiveness.

Chemical base consumption could be fully eliminated through membrane electrolysis using electricity and separation of fatty acids, as was demonstrated by Andersen et al. (2015). They fermented thin stillage into VFAs and MCFAs using a membrane electrolysis system and no chemical pH control. Such system, however, consumes a substantial amount of energy. Based on their experiments, they estimated a power input of 2 kWh per produced kg COD_fatty_ _acids_. The developed two-stage system in the present study, at long HRT, consumed 0.22 kg NaOH per produced kg COD_fatty_ _acids_. Assuming an electricity consumption of 3 kWh/kg NaOH via the chloralkali process (Euro Chlor, 2010), the two-stage system would require less energy for pH control per produced kg COD (0.67 kWh/kg COD_fatty_ _acids_) compared to the in-site membrane electrolysis system. However, whereas the membrane electrolysis system already separated fatty acids from the fermentation broth, the two-stage system would require an additional product separation step to be comparable in electricity consumption.

Effective MCFA production from organic waste can be further developed by reducing the need for chemicals and/or electricity. Possibly, also water-use can be reduced too since the two-stage system used a substantial amount of water to dilute the waste before use as fermentation feed. The presented scenario in the present study, as well as the mentioned alternative scenarios (e.g., Lim et al., 2008; Andersen et al., 2015; Xu et al., 2017) can be optimized and assessed with a case-specific life cycle assessment to make a complete justified discussion on the total environmental impact.

### Future outlook

In a previous study, it was shown that EEO in a chain elongation process can be limited to 15.9% of total ethanol consumption by reducing the CO_2_ loading rate to 0.5 L_CO2_/L/d (Roghair et al., 2018). In the present study, an alternative strategy to suppress EEO is provided: by applying a long HRT, EEO was limited to a similar extent (14.7%). A major advantage of this strategy is that the n-caproate concentration can become much higher. By further increasing the HRT possibly higher n-caproate concentrations can be reached. This could potentially lead to even more limited rate of EEO and thus base consumption in the chain elongation process. Of course, this is only feasible when the process is not limited in substrates (ethanol and VFAs) and CO_2_.

Based on this study and the availability of 88 million ton wet food waste per year in the European Union (Stenmarck et al., 2017), the developed process has the prospects to produce 29 million ton MCFAs per year. This was calculated using the ratios in Figure 2 (at long HRT) and by assuming that wet food waste has the same VS content as in this study; calculations are shown in the supplementary material. The required ethanol (~311 million barrels) would be 37% of total annual global ethanol production (~844 million barrels, Renewable Fuels Association, 2017). The required electrical power for NaOH production (38.4 tWh) would be 13% of total annual wind energy production in Europe (305.8 tWh, International Energy Agency, 2015).

After selective extraction of the MCFAs, they can be further processed into a fuel (i.e., a mixture of hydrocarbons) via Kolbe electrolysis (Khor et al., 2017; Urban et al., 2017). Assuming no losses during extraction and a theoretical efficiency of 0.61 g_hydrocarbons_/g_MCFA_ in the Kolbe electrolysis process and a fuel density of 0.73 g/cm^3^, it is possible to produce ~202 million barrels of fuel per year. This is approximately 7% of the nowadays annual aviation fuel consumption (~2,710 million barrels, International Air Transport Association, 2017).

## Author contributions

MR planned and performed the experiments, analyzed the results, and wrote the manuscript. YL assisted in experimental work and analytical work. DS assisted in the design of the study and revised the manuscript. MB, RW, and CB revised the manuscript. All authors read and approved the final manuscript.

### Conflict of interest statement

The authors declare that the research was conducted in the absence of any commercial or financial relationships that could be construed as a potential conflict of interest.

## Footnotes

^1^^a^Based on 5–6 m^3^/m^3^/d with a methane content of 50–70% Syngellakis, 2015

## Abbreviations

VFAs: Volatile Fatty Acids
MCFAs: Medium Chain Fatty Acids
HRT: Hydraulic Retention Time
AFW: Acidified Food Waste
FW: Food Waste
NaOH: Sodium Hydroxide
EEO: Excessive Ethanol Oxidation
VS: Volatile Solids
VSS: Volatile Suspended Solids
TS: Total Solids
COD: Chemical Oxygen Demand
UASB: Upflow Anaerobic Sludge Blanket.
