# Supplementary material for: Development of an Effective Chain Elongation Process From Acidified Food Waste and Ethanol Into n-Caproate
Source: Front Bioeng Biotechnol. 2018 Apr 27;6:50. doi: 10.3389/fbioe.2018.00050 (PMC5934429; doi:10.3389/fbioe.2018.00050)
Supplement: Supplementary file 1 [file Data_Sheet_1.pdf]

Supplementary material

Development of an Effective Chain Elongation Process From Acidified Food Waste and Ethanol Into n-Caproate

Mark Roghair [a], Yuchen Liu [a], David P.B.T.B. Strik [a,], Ruud A. Weusthuis [b], Marieke E. Bruins [c], Cees J.N. Buisman [a]*

[a] Sub-department of Environmental Technology, Wageningen University & Research, Bornse Weilanden 9, 6708 WG, Wageningen, the Netherlands
[b] Bioprocess Engineering, Wageningen University & Research, Droevendaalsesteeg 1, 6708 PB, Wageningen, the Netherlands
[c] Wageningen Food & Biobased Research, Wageningen University & Research, Bornse Weilanden 9, 6708 WG, Wageningen, the Netherlands

* Corresponding author: E-mail: david.strik@wur.nl; tel +31 317 483 447

Table S1. Mean steady state values at long HRT (day 28 - 58)

Compound	Concentration<br>[mmol·L^{-1}]	Rate<br>[mmol·L^{-1}·d^{-1}]	Selectivity<br>[mol e %]	Selectivity<br>[mol C %]
Ethanol	61.4 ± 23.6	-130.8 ± 6.9	N.A.	N.A.
Propanol	1.1 ± 1.0	0.3 ± 0.3	0.3	0.2
Acetate	70.6 ± 12.9	-15.9 ± 2.9	N.A.	N.A.
Propionate	6.0 ± 0.8	-4.5 ± 0.1	N.A.	N.A.
Isobutyrate	5.4 ± 0.5	-0.4 ± 0.1	N.A.	N.A.
n-Butyrate	64.1 ± 7.3	-9.9 ± 1.6	N.A.	N.A.
Isovalerate	3.8 ± 0.3	-0.1 ± 0.1	N.A.	N.A.
n-Valerate	16.2 ± 4.4	3.0 ± 1.2	3.9	4.0
Isocaproate	0.9 ± 0.2	0.2 ± 0.1	0.4	0.4
n-Caproate	201.3 ± 8.7	47.1 ± 3.1	76.5	76.2
n-Heptanoate	4.3 ± 1.7	1.1 ± 0.4	2.1	2.0
n-Caprylate	4.7 ± 1.0	1.2 ± 0.3	2.6	2.5
CO_2	52.7 ± 20.1 % *	-22.1 ± 1.8	N.A.	N.A.
CH_4	37.7 ± 14.7 % *	12.8 ± 2.6	5.2	3.5
H_2	<0.1% *		N.A.	N.A.
Unidentified			9.1	11.2

Table S2. Mean steady state values at short HRT (day 79 - 103)

Compound	Concentration<br>[mmol·L^{-1}]	Rate<br>[mmol·L^{-1}·d^{-1}]	Selectivity<br>[mol e %]	Selectivity<br>[mol C %]
Ethanol	437.3 ± 34.0	-267.6 ± 23.5	N.A.	N.A.
Propanol	0.7 ± 0.9	0.7 ± 0.9	0.4	0.3
Acetate	122.1 ± 8.6	-13.1 ± 8.3	N.A.	N.A.
Propionate	15.5 ± 1.0	-9.2 ± 1.5	N.A.	N.A.
Isobutyrate	6.2 ± 0.4	-0.9 ± 0.4	N.A.	N.A.
n-Butyrate	122.8 ± 11.3	18.3 ± 12.4	11.0	12.1
Isovalerate	4.0 ± 0.3	-0.5 ± 0.3	N.A.	N.A.
n-Valerate	9.4 ± 1.1	4.3 ± 0.9	3.2	3.4
Isocaproate	0.4 ± 0.1	0.4 ± 0.1	0.4	0.4
n-Caproate	60.7 ± 7.8	48.1 ± 7.5	44.6	46.0
n-Heptanoate	0.6 ± 0.1	0.6 ± 0.1	0.6	0.6
n-Caprylate	0.5 ± 0.1	0.5 ± 0.1	0.7	0.7
CO_2	5.4 ± 3.0 % *	-37.2 ± 0.1	N.A.	N.A.
CH_4	77.0 ± 42.0 % *	43.8 ± 2.5	10.2	7.0
H_2	<0.1 % *		N.A.	N.A.
Unidentified			29.0	29.5

* Concentrations of gaseous compounds (CO_2, CH_4, H_2) are shown as percentage in the headspace at 1 atm

Table S3. Equations to determine how much food waste (FW; expressed in g volatile solids), ethanol (EtOH) and sodium hydroxide (NaOH) would be consumed to yield 1000 g MCFAs in the two-stage system

Compound	Unit	Equation
Food waste (FW)	g VS	$FW = \left(\frac{1000\ \text{g MCFA}}{\overline{MW}_{MCFA}} \cdot \bar{C}_{MCFA} - Y_{\frac{MCFA}{FW}} \cdot \text{FW}\right) \cdot \left\lvert\frac{\text{r}_{VFA}}{r_{MCFA}}\right\rvert \cdot \frac{1}{Y_{\frac{VFA}{FW}}}$
Ethanol (EtOH)	g	$EtOH = \left(\frac{1000\ \text{g MCFA}}{\overline{MW}_{MCFA}} \cdot \bar{C}_{MCFA} - Y_{\frac{MCFA}{FW}} \cdot \text{FW}\right) \cdot \left\lvert\frac{\text{r}_{EtOH}}{r_{MCFA}}\right\rvert \cdot \frac{MW_{EtOH}}{C_{EtOH}}$
NaOH (Hydrolysis and acidogenesis stage)	g	$NaOH = FW \cdot \frac{\Delta NaOH}{\Delta FW}$
NaOH (Chain elongation stage)	g	$NaOH = EtOH \cdot \frac{r_{NaOH}}{r_{EtOH}}$

Table S4. Parameters that we used as input for the equations in Table S3 to determine how much food waste (in g VS), ethanol (EtOH) and sodium hydroxide (NaOH) would be consumed to yield 1000 g MCFAs in the two-stage system.

Parameter description	Symbol	unit	Value HRT = 4 d	Value HRT = 1 d
Hydrolysis and acidification stage				
VFA yield on FW	$Y_{\frac{VFA}{FW}}$	mol C/g VS	$35.14 \cdot 10^{-3}$	$35.14 \cdot 10^{-3}$
MCFA yield on FW	$Y_{\frac{MCFA}{FW}}$	mol C/g VS	$3.39 \cdot 10^{-3}$	$3.39 \cdot 10^{-3}$
Consumed NaOH per consumed FW	$\frac{\Delta NaOH}{\Delta FW}$	g/g VS	0.32	0.32
Chain elongation stage [i]				
Consumed VFAs per produced MCFAs	$\frac{\mathrm{r}_{VFA}}{r_{MCFA}}$	mol C/mol C	0.29	0.20
Consumed ethanol per produced MCFAs	$\frac{\mathrm{r}_{EtOH}}{r_{MCFA}}$	mol C/mol C	0.87	1.83
Average carbon atoms per produced MCFAs	$\bar{C}_{MCFA}$	C-atoms/mol	6.07	6.03
Average molar weight of produced MCFAs	$\overline{MW}_{MCFA}$	g/mol	117.13	116.61
Consumed NaOH per consumed ethanol	$\frac{r_{NaOH}}{r_{EtOH}}$	g/g	0.30	0.30
Constants				
Molar weight of ethanol	MW_{EtOH}	g/mol	46.07	46.07
Carbon atoms per mole ethanol	C_{EtOH}	C-atoms/mol	2	2

[i] Values are based on steady state data

Future Outlook: Calculations

MCFA production from wet food waste:

88 mln ton wet food waste / yr · (13.8 g VS / 100 g wet food waste)[i] · (1000 g MCFAs / 419 g VS)[i] = 28.9 mln ton MCFAs / yr

Required ethanol for MCFA production:

29 mln ton MCFAs / yr · 1000 · (1013 g ethanol / 1000 g MCFA)[i] / (0.789 kg / L) / (119.2 L / barrel) = 311.1 mln barrels ethanol / yr

Required NaOH for MCFA production:

29 mln ton MCFAs / yr · ((136 g NaOH + 307 g NaOH) / 1000 g MCFA)[i] = 12.8 mln ton NaOH / yr

Required electricity to produce NaOH for MCFA production:

12.8 mln ton NaOH / yr · (3 kWh / kg NaOH)[ii] = 38.4 · 1000 mln kW h / yr = 38.4 TW h / yr

Fuel production from MCFAs:

29 mln ton MCFAs / yr · 1000 · (0.61 g hydrocarbon / g MCFA)[iii] / (0.73 kg / L)[iii] / (119.2 L / barrel) = 202 mln barrels fuel / yr

[i] Values based on this study (Figure 2)
[ii] Electricity consumption through the Chlorialkali process
[iii] Assumption / theoretical efficiency
